# Neutrophil extracellular trap induced by HMGB1 exacerbates damages in the ischemic brain

**DOI:** 10.1186/s40478-019-0747-x

**Published:** 2019-06-10

**Authors:** Seung-Woo Kim, Hahnbie Lee, Hye-Kyung Lee, Il-Doo Kim, Ja-Kyeong Lee

**Affiliations:** 1 Department of Biomedical Sciences, Inha University School of Medicine, Inchon, Republic of Korea; 2 Medical Research Center, Inha University School of Medicine, inha 100, Nam-Gu, Inchon, 22212 Republic of Korea; 3 Department of Anatomy, Inha University School of Medicine, inha 100, Nam-Gu, Inchon, 22212 Republic of Korea

**Keywords:** HMGB1, NETosis, Inflammation, MCAO, Permanent ischemia

## Abstract

It has been reported that neutrophil extracellular traps (NETs) play important roles in non-infectious diseases. In ischemic stroke, neutrophils infiltrate damaged brain tissue soon after injury and aggravate inflammation. Using a rat permanent MCAO model, we showed citrullinated histone H3^+^ (CitH3, a marker of NETosis) induction in neutrophils in leptomeninges and in peripheral blood soon after MCAO. Entry of CitH3^+^ cells occurred through leptomeninges after 6 h of MCAO and these cells were observed in cerebral cortex from 12 h and subsequently in striatum. It is interesting to note that CitH3^+^ induction began in circulating neutrophils before they migrated to brain parenchyma and they were detected as intact or lysed form. High mobility group box 1 (HMGB1), a danger associated molecular pattern (DAMP) molecule, was accumulated massively in serum after permanent MCAO and plays a critical role in CitH3 inductions in neutrophils in brain parenchyma and in peripheral blood. Both the all-thiol and disulfide types of HMGB1 induced CitH3 via their specific receptors, CXCR4 and TLR4, respectively. Importantly, HMGB1 not only induced NETosis but was included as a part of the extruded NETs, and contribute to NETosis-mediated neuronal death. Therefore, it would appear a vicious cycle exists between neuronal cell death and NETosis and HMGB1 mediates detrimental effects exerted by this cycle. When NETosis was suppressed by a PAD inhibitor in MCAO animals, delayed immune cell infiltrations were markedly suppressed and damages in blood vessels were significantly mitigated. The study shows NETosis with the involvement of HMGB1 as a mediator in a vicious cycle aggravates inflammation and subsequent damage in the ischemic brain.

**Electronic supplementary material:**

The online version of this article (10.1186/s40478-019-0747-x) contains supplementary material, which is available to authorized users.

## Introduction

Neutrophils are the most abundant white blood cells and are rapidly recruited to infected sites to fight against pathogen attack. Neutrophils employ several strategies to kill and process invading bacteria and to modulate inflammation, which involve the productions of antibacterial peptides, reactive oxygen species (ROS), and proinflammatory mediators [10, 22, 25, 39]. Recently, it was reported neutrophil extracellular traps (NETs) are formed with projections of decondensed chromatin and granular contents and that they constitute an effective antimicrobial defense mechanism [8, 11]. Although NETs play important roles in host defense under infection, NET formation has recently been implicated in various sterile inflammatory conditions and in the perpetuation of inflammation and tissue damage. For example, extensive NET formation has been shown to aggravate autoimmune diseases, such as, systemic lupus erythematosus and lupus nephritis [23, 38] and various sterile inflammatory conditions such as, atherosclerosis, venous thrombosis, lung injury, and tumor metastasis [2, 7, 18, 34].

Neutrophils infiltrated damaged brain tissue early in various pathological conditions of the central nervous system (CNS), such as ischemic stroke, hemorrhage, and trauma [29, 40, 45]. Infiltrating leukocytes produce pro-inflammatory cytokines, matrix metalloproteinases (MMPs), nitric oxide (NO), ROS, and other cytotoxic molecules, which accelerate brain damage [1, 5]. Moreover, NETosis induction has been reported in various pathological conditions of brain, and in particular, citrullinated histone H3 (CitH3) induction in neutrophils (a marker of NETosis) has been reported in an animal model of stroke [27] and areas affected by thrombus in acute ischemic stroke patients [36]. NET formation has also been reported in a mouse model of Alzheimer’s disease [44] and serum MPO-DNA complex levels were reported to be significantly elevated in multiple sclerosis patients [33].

Recently, high mobility group box 1 (HMGB1; a danger associated molecular pattern (DAMP) molecule) was suggested as a candidate NETosis inducer in non-infectious diseases [21, 30]. In a liver ischemia/reperfusion injury (I/R) model, recombinant HMGB1 protein treatment increased CitH3 expression and NET formation via TLR4/9 [9] and HMGB1 induced formation of prothrombotic NETs in deep venous thrombosis animal model [31]. In previous studies, we observed HMGB1 is massively released into the extracellular space after ischemic brain injury and aggravates inflammation in a paracrine and autocrine manner [13–15]. HMGB1 contains three conserved redox-sensitive cysteines that determine its functional activity in different pathological contexts. Depending on oxidation state, the following three types of HMGB1 are present: 1) disulfide HMGB1, which activates TLR4/MD-2 complex and functions as a cytokine [42]; 2) all-thiol HMGB1, which recruits neutrophils and monocytes to inflammatory sites by complexing with CXCR12 and binds CXCR4 [37]; and 3) (terminally) oxidized HMGB1 which cannot bind CXCR4 or TLR4 [41]. Although importance of TLR4 and RAGE in the HMGB1-mediated induction of NETosis has been reported [9, 31], the differential functions of different types of HMGB1 have not been previously studied. In the present study, we examined the temporal and spatial progress of NETosis and its importance in the ischemic brain using a rat model of permanent MCAO. In particular, we investigated the importance of HMGB1 as a NETosis inducer in the ischemic brain and the differential functions of all-thiol and disulfide HMGB1 and their signaling pathways.

## Materials and methods

### Surgical procedure

The animal protocol used was reviewed beforehand and approved by the INHA University-Institutional Animal Care and Use Committee (INHA-IACUC) with respect to ethicality (Approval Number INHA-141124-337-2). All procedures concerning animals were in accord with the Guide for the Care and Use of Laboratory Animals published by the National Institute of Health (2010) and with ARRIVE (Animal Research: Reporting of In Vivo Experiments) guideline [12]. Male Sprague-Dawley (SD) rats (8 weeks) weighing 230–250 g were purchased from Orient Bio Inc. (Gyeonggi, South Korea) and housed under diurnal lighting conditions with ad libitum access to food and tap water for a week before experiments. Male SD rats (9 weeks) were anesthetized with 5% isoflurane in a 30% oxygen/70% nitrous oxide mixture delivered through a close-fitting facemask during surgery. The permanent MCAO-induced focal cerebral ischemia model used was generated as described previously [20]. Briefly, the right common carotid artery (CCA), internal carotid artery (ICA), and external carotid artery (ECA) were exposed through a midline incision of the neck, and the ECA and CCA were ligated with silk sutures and the ICA was temporarily clipped. A monofilament nylon suture (4–0; AILEE, Busan, Korea) was then inserted into the ICA and slowly advanced from the lumen of the ICA to the MCA; this suture was left in place until sacrifice. All procedures were finished within 15 min. Sham controls underwent CCA, ICA, and ECA exposure only. During the procedure, left femoral arteries were cannulated to obtain blood samples, which were analyzed for pH, PaO_2_, PaCO_2_, and blood glucose concentration (I-STAT; Sensor Devices, Waukesha, WI). Laser Doppler flowmeter (Periflux System 5000; Perimed, Jarfalla, Sweden) was used to monitor regional cerebral blood flow (CBF) and relative CBF during experiment, and a thermoregulated heating pad and a heating lamp were used to maintain a rectal temperature of 37.0 ± 0.5 °C during surgeries.

### HMGB1 a box or anti-HMGB1 antibody administration

HMGB1 A box (50 μg/kg; HMGbiotech, Milano, Italy; HM-012) was administered intravenously in 0.3 ml PBS after 3 h of pMCAO. Anti-HMGB1 antibody (Santa Cruz Biotechnology, Santa Cruz, CA) was also administered intravenously at the dose of 200 μg/kg after 3 h of pMCAO. Animals in the sham group were injected with 0.3 ml of PBS intravenously at 3 h after surgery.

### Immunohistochemistry

Animals were sacrificed at the indicated times after surgery. Brains were isolated and fixed with 4% paraformaldehyde (PFA; Sigma Aldrich, St. Louis, MO) by transcardiac perfusion and then stored in the same solution overnight at 4 °C. Brain sections (40 μm) were prepared using a vibratome and immunological staining was performed using a previously described floating method [13]. The sections were preincubated in blocking solution containing 5% FBS, 5% horse serum, and 2% BSA in PBS. Primary antibodies were diluted 1:500 for anti-ionized calcium binding adaptor molecule-1 (Iba-1) (Wako Pure Chemicals, Osaka, Japan). After washing with PBS containing 0.1% Triton X-100, sections were incubated with anti-rabbit IgG (Vectorlab, Peterborough, UK) in PBS for 1 h at room temperature and visualized using the HRP/3,3-diaminobenzidine (DAB) system and observed under a fluorescence microscope (Axioplan 2; Zeiss, Oberkochen, Germany).

### Immunofluorescent staining

Polymorphonuclear leukocytes (PMNs) were prepared from peripheral blood or CSF and a cytospin was used to immobilize them on slides. After cytospin centrifugation, slides were fixed for 15 min. Polymorphonuclear leukocytes prepared from bone marrow (PMNs-BM) were cultured in 24 well plate and fixed for 15 min. Both PMNs and PMNs-BM were blocked with 1% normal goat serum and incubated overnight with anti-CitH3 antibody (ab18956–100; Abcam, Cambridge, UK) or anti HMGB1 antibody (ab672; Abcam, Cambridge, UK) at 4 °C. Brain sections were prepared as described in immunohistochemistry. The sections were preincubated in blocking solution containing 5% FBS, 5% horse serum, and 2% BSA in PBS. Primary antibodies for anti-CitH3 (ab18956–100; Abcam, Cambridge, UK), anti-Ly6g-FITC (ab2949, Abcam, Cambridge, UK), anti-MPO-FITC (ab18956, Abcam, Cambridge, UK), anti-RECA (MCA970GA, Bio-rad, Kidlington, UK), and anti-lamin (ab78946; Abcam, Cambridge, UK) were diluted 1:200. For double immunostaining, rhodamine anti-rabbit IgG (Jackson ImmunoRes Lab, West Grove, PA) was used as the secondary antibody and incubated for 1 h at room temperature. Brain sections or cytospin slides were counterstained with DAPI (4′,6-diamidino-2-phenylindole; Sigma Aldrich, St. Louis, MO) to visualize nuclei, and observed under a fluorescence microscope (Axioplan 2; Zeiss, Oberkochen, Germany). The numbers of CitH3 positive cells in 0.16 mm^2^ (0.4 × 0.4 mm) were scored and vessel length and density were analyzed using the AngioTool Software (National Cancer Institute, Gaithersburg, MD, USA).

### H & E staining

Brain tissues were fixed overnight in 4% paraformaldehyde (PFA), embedded in paraffin, and cut into 5 μm sections using a microtome. Deparaffinized sections were stained with hematoxylin and eosin (H&E) and observed under a light microscope (Axioplan 2; Zeiss, Oberkochen, Germany).

### TTC staining

Rats were sacrificed at 12 h after MCAO and whole brains were dissected coronally into 2-mm slices using a metallic brain matrix (RBM-40000, ASI, Springville, UT). Slices were immediately incubated in saline containing TTC (2, 3, 5-triphenyl tetrazolium chloride, 2%) for 15 min at 37 °C and then stored in 4% PFA.

### Immunoblotting

Brain tissues or PMNs on cytospin slides were washed twice with cold PBS and lysed in RIPA buffer containing 50 mM Tris-HCl (pH 7.4), 0.5% Triton X-100, 0.5% NP-40, 0.25% sodium-deoxycholate, 150 mM NaCl, and complete Mini protease inhibitor cocktail tablets (1 tablet in 10 ml) (Roche, Basel, Switzerland). Lysates were centrifuged for 15 min at 14000 rpm at 4 °C and supernatants were loaded into 10–12% SDS PAGE gels. Primary antibodies for anti-CitH3 (ab18956–100; Abcam, Cambridge, UK), anti-HMGB1 (ab67282; Abcam, Cambridge, UK), and anti-GAPDH (2118; Cell Signaling Technology, Danvers, MA) were diluted 1:2000–10,000. The signals were detected using a chemiluminescence kit (Merck Millipore, Darmstadt, Germany).

### Preparation of blood and serum

Blood samples were collected via cardiac puncture procedure using 23G syringe without thoracotomy. For serum samples, blood samples (1 ml) were left for 30 min at room temperature. To remove clot, the blood samples were centrifuged for 15 min at 2000 g at 4 °C. and supernatant was transferred immediately to ice-cold new tube. The sample was divided into 100 ul aliquots and stored in EDTA-coated vacutainer (BD Bioscience, Franklin Lakes, NJ).

### Isolation of circulating neutrophils

Neutrophil was isolated from rat blood using Histopaque (Sigma Aldrich, St. Louis, MO) gradients as previously described [30]. Briefly, Histopaque 1077 (3 ml) was layered on Histopaque 1119 (3 ml) in 15 ml tube and rat blood (4 ml) was carefully placed on the top of the Histopaque mixture, which formed a three-step gradient (Histopaque 1119/Histopaque1077/blood). The tube was then centrifuged at 400 g for 30 min using a swinging rotor. The first ring, which contained mononuclear cells was slowly aspirated, and the second ring was transferred to another 15 ml tube containing PBS-BG (phosphate buffered solution, 0.1% bovine serum albumin, and 10% glucose) and centrifuged at 1500 g for 10 min. The pellet so obtained was suspended in 3 ml of PBS-BG, placed on Histopaque-1119 (3 ml), and centrifuged at 1500 g for 10 min at 4 °C. The ring that included neutrophils was then suspended in RPMI (Gibco BRL, Gaithersburg, MD) containing 1% FBS.

### Primary neuron culture

Mixed cortical cells were obtained from embryonic day 15.5 (E15.5) mouse cortices and cultured as described in a previous report [16]. Dissociated cortical cells were plated at a density of five hemispheres per 24-well poly-d-lysine (100 μg/ml) and laminin (100 μg/ml)-coated plate (~ 4 × 10^5^ cells per well). Cultures were maintained without antibiotics in MEM containing 5% FBS and 5% horse serum. At day 7 in vitro (DIV 7), when astrocytes had reached confluence underneath neurons, cytosine arabinofuranoside (ara-C, Sigma Aldrich, St. Louis, MO) was added to a final concentration of 10 μM, and culture was maintained for 2 d to halt microglial growth. Fetal bovine serum (FBS) and glutamine were not supplemented from DIV 7, and the medium was changed twice weekly after DIV 7. Cultures were used at DIV 12–14.

### Treatment of neutrophils with NMDA conditioned media (NCM) or of neurons with NCM-treated neutrophil

Primary cortical neurons (4 × 10^5^/well) were treated with MEM (21 mM glucose) containing 300 μM NMDA (Sigma, St. Louis, MO) for 30 min, washed twice with PBS, cultured in fresh MEM for 4 h. Neutrophils were then treated with this NMDA-conditioned medium (NCM, 1.6 × 10^6^ /4 wells) for the indicated times. NCM-treated neutrophils (5 × 10^5^/well) were co-cultured with primary cortical neurons using a transwell co-culture device (pore size 3 μm) (SPL Lifescience, Gyeonggi, South Korea). Neutrophils were pretreated with AMD3100 (Sigma, St. Louis, MO), TLR-IN-C34 (Sigma, St. Louis, MO), or Cl-amidine (PAD inhibitor; Merk Millipore, Burlington, MA) for 30 min before NCM treatment. NCM was preincubated with anti-HMGB1 antibody or HMGB1 A box (HMGbiotech, Milano, Italy; HM-012) for 30 min and then treated to neutrophils. For propidium iodide (PI) staining, PI (1 μg/ ml) was added to co-culture and incubation continued for 30 min. Primary cortical neurons were then fixed in 4% PFA for 20 min and fluorescence was visualized under a fluorescence microscope (Axioplan 2; Carl Zeiss, Oberkochen, Germany). The numbers of PI positive cells in 250 μm × 250 μm were counted and.

### Statistical analysis

Sample sizes for animal experiments were determined by using power calculation software (http://www.gpower.hhu.de/) and the levels of significance are 5% with 80% power as a minimum. Differences in the parameters was performed by using analysis of variance (ANOVA) followed by the Newman-Keuls test. For a non-parametric statistics test, Kruskal-Wallis H non parametric test was performed, followed by Tukey’s test on SPSS package 18. Simple comparisons for histological data were carried out using Student’s t-tests. All results are presented as means±SEMs, and statistical difference was accepted at *P*- value < 0.05.

## Results

### Spatio-temporal profiles of CitH3 induction in brain after MCAO

**Fig. 1:**
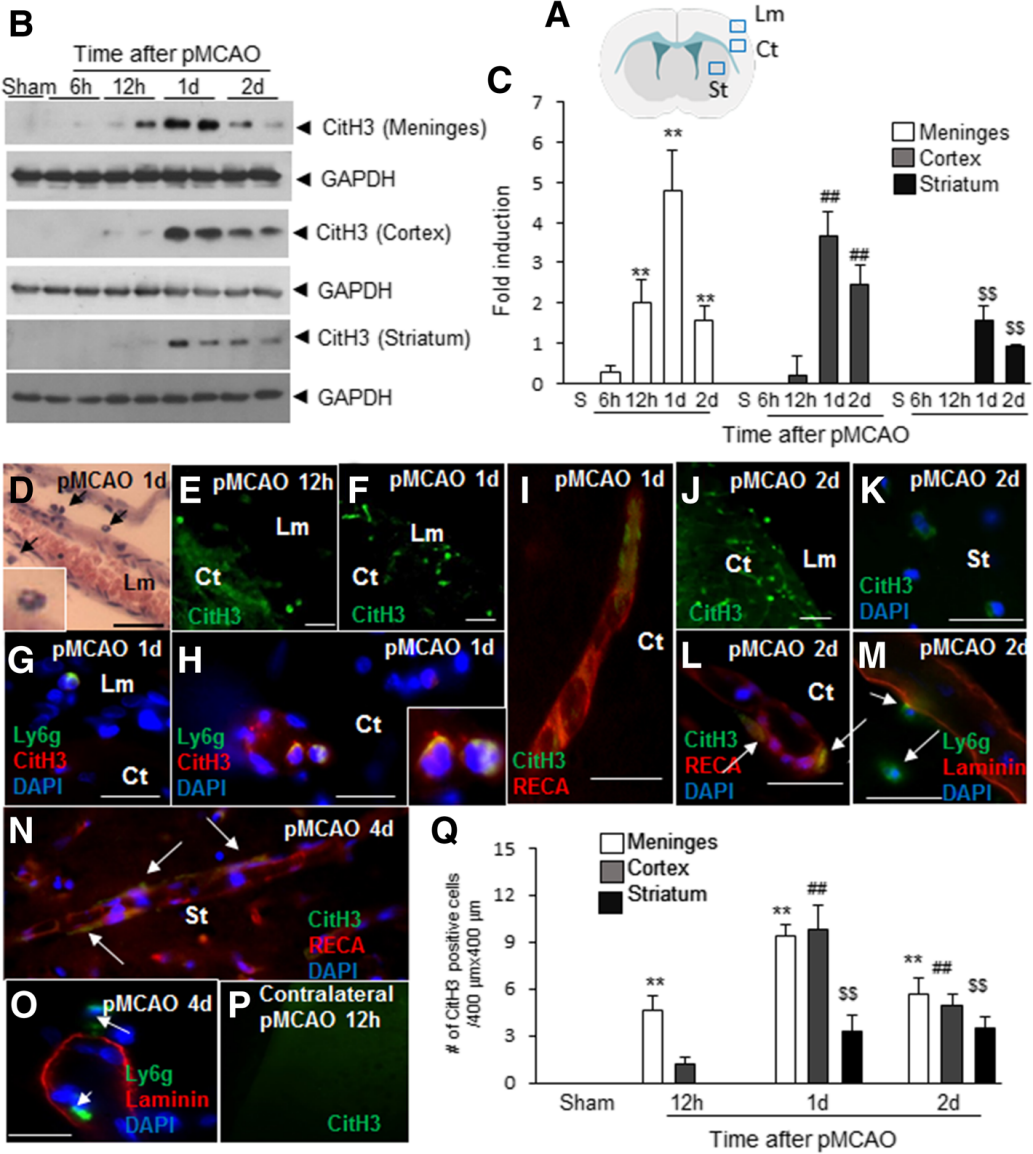
Elevation of CitH3 levels in ischemic brains after MCAO. **a** Three brain regions used in the present study are indicated as follows, leptomeninges (Lm), cortex (Ct), and striatum (St). **b**-**c** Levels of CitH3 were examined in Lm, Ct, and St after 6, 12, 24, and 48 h of MCAO by immunoblotting (GAPDH was used as a loading control). Representative images are presented and results are presented as means±SEMs (*n* = 4). **d** Coronal brain sections were prepared after 24 h of MCAO and H&E stained. **e**-**l** Coronal brain sections were prepared after 12 h, 24, and 48 h of MCAO as indicated and immunofluorescent staining was conducted with anti-CitH3 antibody (**e,f,j**, and **p**) or with anti-CitH3, anti-Ly6g antibody, anti-RECA, anti-laminin, or DAPI (**g-i** and **k-o**). The photographs shown are representative of three independent experiments. The insets in D and H are high magnification photographs of each image. Scale bars in D-F and J represent 50 μm and those in G-I and K-O represent 20 μm. **q** CitH3^+^ cells in leptomeninges, cortex, and striatum (400 μm × 400 μm) were counted and results are presented as means±SEMs (*n* = 7–9 from 3 to 5 animals). **p < 0.01, ^##^*p* < 0.01, ^$$^*p* < 0.01 versus Sham-control

Levels of CitH3 were examined in leptomeninges, cerebral cortices, and striata in the ipsilateral hemisphere of the ischemic brain by immunoblotting (Fig. 1a). CitH3 induction begun after 6 h of MCAO in the leptomeninges, in which it peaked at ~ 24 h (Figs. 1b and c). In ischemic cortices, marked CitH3 induction was observed after 24 h of MCAO, and thereafter it slowly decreased (Figs. 1 b and c). CitH3 levels were also elevated in striata at 24–48 h but to lesser degrees (Figs. 1 b and c). H&E staining of brain sections revealed that numerous cells with a multi-lobular nucleus were localized in leptomeninges after 24 h of MCAO, indicating neutrophil accumulation (Fig. 1d). Immunofluorescent staining with anti-CitH3 antibody showed numbers of CitH3^+^ cells were significantly increased in leptomeninges and cortical parenchyma at 12 h and further increased at 24 and 48 h (Figs. 1 e, f, j, and p). Triple fluorescent staining with anti-CitH3 antibody, anti-Ly6g antibody (a neutrophil marker) [4], plus DAPI confirmed the CitH3^+^ cells observed in parenchyma are neutrophils and they were observed both in parenchyma (Figs. 1 g, h, and k) and in intra- or perivascular regions (Figs. 1 h, i, and l-o). Interestingly, CitH3^+^ immunoreactivity was observed in non-lytic cells in the intravascular region after 24 h of MCAO; these cells were flattened and adhered to the luminal sides of endothelial walls (Fig. 1i). After 48 h of MCAO, CitH3^+^ cells were observed in intravascular, perivascular, and parenchymal regions (Figs. 1l and m). Fewer numbers of CitH3^+^ cells were detected in striatum at more delayed time points (Figs. 1n and o). Notably, no CitH3^+^ cells were detected in contralateral hemispheres (Fig. 1p). CitH3^+^ cell counts in ischemic brains (Fig. 1q) indicated first CitH3^+^ cell entry occurred through leptomeninges at ~ 12 h and then they were observed in cerebral cortices and in striata after 24 h of pMCAO.

### Rapid inductions of CitH3 in PMNs in peripheral blood and CSF after MCAO

**Fig. 2:**
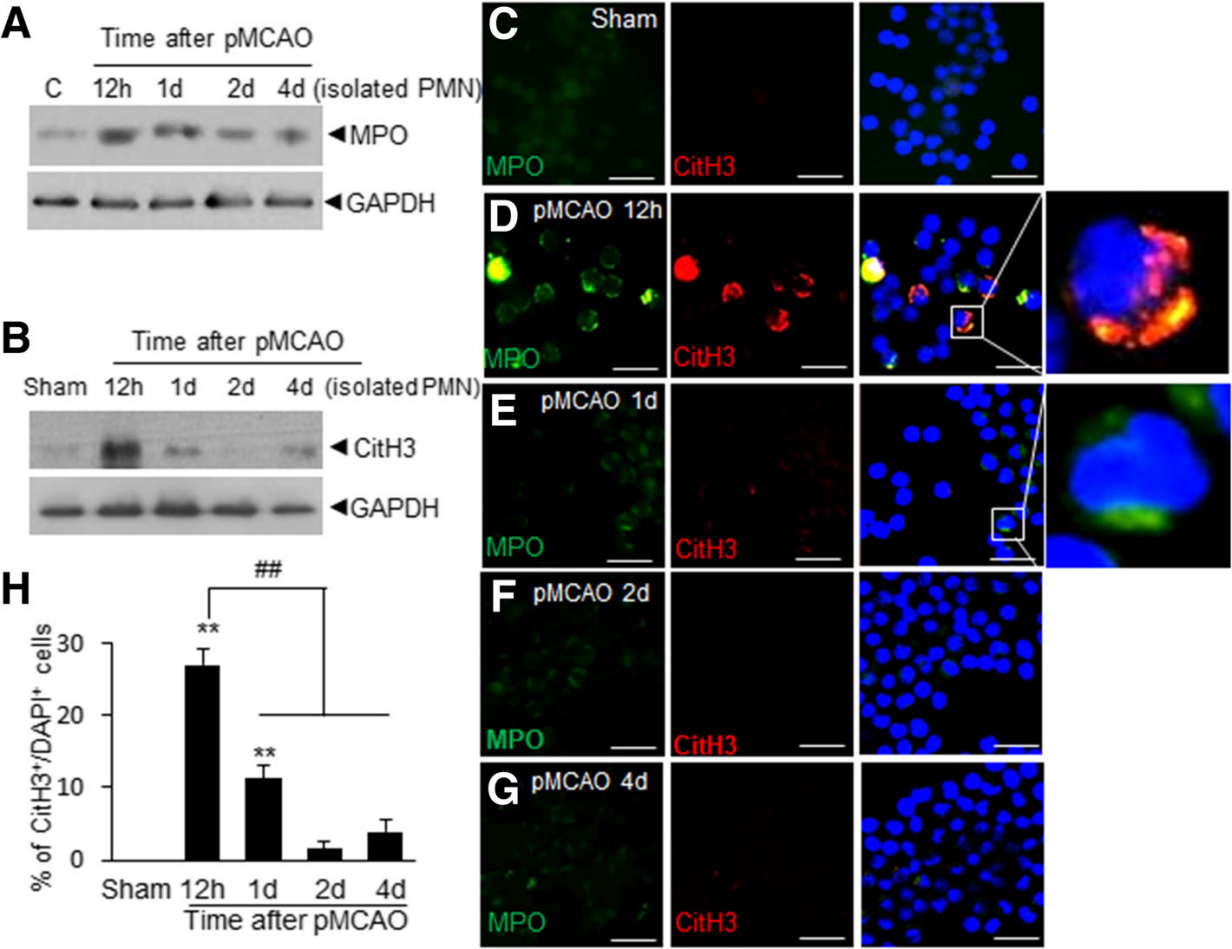
CitH3 levels and numbers of CitH3^+^ cells in peripheral blood after MCAO. Neutrophils were purified from peripheral blood after 12 h, 1 d, 2 d, and 4 d of MCAO. **a-b** Levels of MPO and CitH3 were examined in isolated neutrophils by immunoblotting (GAPDH was used as a loading control). **c** Triple immunofluorescent staining was conducted using anti-CitH3 antibody, anti-MPO antibody, plus DAPI. Scale bars in C-G represent 50 μm. The images in right panel are high magnification photographs of each image indicated as white box. **d** Ratio of CitH3^+^/DAPI^+^ cells are presented as means±SEMs (*n* = 4–8 from 4 to 8 animals). **p < 0.01 versus Sham-control

Observation of the presence of CitH3^+^ cells in the intravascular region prompted us to investigate kinetics of CitH3 induction in peripheral blood neutrophils before extravasation. We isolated circulating neutrophils from peripheral blood of sham- and MCAO-operated rats and accessed CitH3 levels by immunoblotting. When we examined MPO level in circulating neutrophils, it was markedly increased after 12 h of MCAO and then slowly decreased, suggesting that the number of neutrophils increased and their activation occurred in peripheral blood (Fig. 2a). Interestingly, CitH3 level in circulating neutrophils was markedly increased after 12 h of MCAO and then decreased abruptly, indicating a rapid induction of NETosis in peripheral neutrophils (Fig. 2b). Triple immunofluorescent staining of purified neutrophils with anti-MPO, anti-CitH3 antibodies, plus DAPI confirmed that in sham-operated animals, no CitH3^+^ cells were observed, however, CitH3^+^ cell numbers markedly increased at 12 h and the percentage of CitH3-expressing MPO^+^ cells among DAPI^+^ cells reached 27.0 ± 2.2% (Figs. 2c, d, and h). After 24 h of MCAO, the ratio of CitH3^+^/DAPI^+^ cells was reduced and this reduced further at 48 h (Fig. 2e, f, and h), which was in-line with our immunoblot results (Fig. 2b). Notably, we also detected CitH3^+^/MPO^+^ double positive cells in CSF at 12 h (Additional file 1: Figure S1). These observations indicate that NETosis began in circulating neutrophils before they migrated to brain parenchyma in ischemic animals.

### Induction of NETosis by HMGB1 in peripheral blood and bone marrow PMNs

**Fig. 3:**
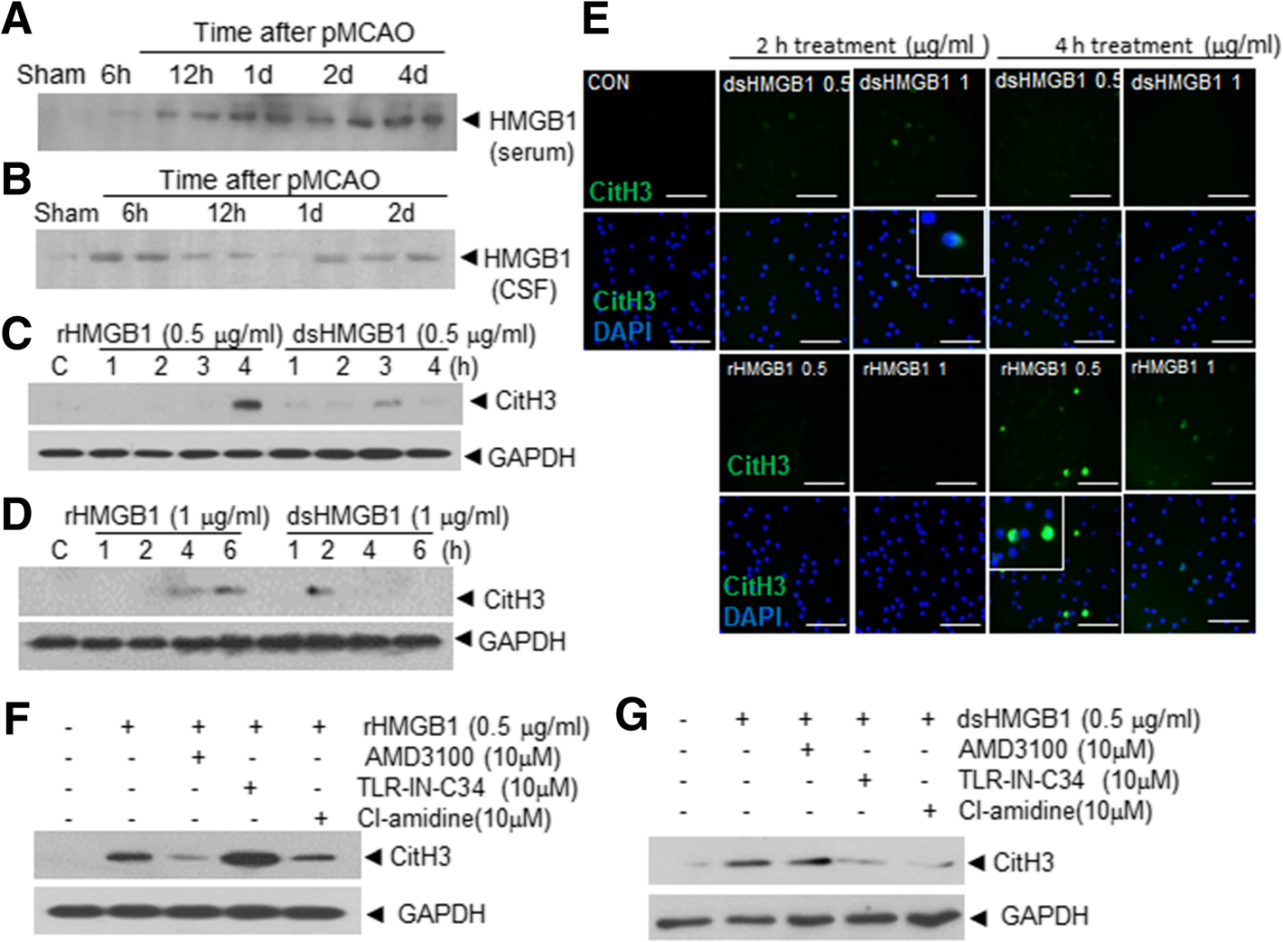
CitH3 inductions in blood PMNs by HMGB1 treatment. **a-b** HMGB1 levels in blood and CSF were determined after 6 h to 4 d of MCAO by immunoblotting. **c-d** CitH3 levels were examined after treating neutrophils isolated from peripheral blood with all-thiol HMGB1 or disulfide HMGB1 (0.5 or 1 μg/ml) for 1, 2, 3, and 4 h or for 1, 2, 4, and 6 h, respectively, by immunoblotting. **e** Blood neutrophils were treated with all-thiol HMGB1 or disulfide HMGB1 (0.5 or 1 μg/ml) for 2 or 4 h and double fluorescent staining was conducted using anti-CitH3 antibody plus DAPI. **f-g** CitH3 levels were examined by immunoblotting after treating blood neutrophils with 0.5 μg/ml of all-thiol HMGB1 (**f**) or disulfide HMGB1 (**g**) for 4 h with or without pre-treating circulating neutrophils with AMD3100 (10 μM), TLR-IN-C34 (10 μM), or PAD inhibitor (10 μM) for 30 min. Scale bars in E represent 100 μm

Various infectious and non-infectious stimuli have been reported to induce NETs, but the stimuli responsible for NET formation during cerebral ischemia remain unknown. HMGB1 is a prototypic DAMP and we previously reported marked HMGB1 accumulation in CSF and serum after transient MCAO, and its aggravation of brain damage via autocrine and paracrine mechanisms [13, 14, 17]. To determine whether HMGB1 induces NETosis in the ischemic brain, we first examined HMGB1 levels in blood and CSF after permanent MCAO. HMGB1 progressively and markedly accumulated in serum after MCAO (Fig. 3a). In addition, a rapid increase in HMGB1 in CSF was observed after 6 h of MCAO and its level was maintained at moderate levels for several days (Fig. 3b). Since cellular sources of extracellular HMGB1 might vary in different pathological states [17, 35], we first examined NETosis-inducing effect of HMGB1 using recombinant protein. Treatment of purified peripheral neutrophils with recombinant HMGB1 showed that both the all-thiol and disulfide types of HMGB1 were able to induce CitH3 significantly (Figs. 3c and d). Dosage and duration tests indicated that these two types had different efficiencies, that is, disulfide HMGB1 at same dosages more rapidly induced CitH3 upregulation and NET formation than the all thiol type, whereas all-thiol HMGB1 induced CitH3 upregulation more strongly than disulfide HMGB1 at the same doses (Figs. 3)c and d. Double immunofluorescent staining of neutrophils with anti-CitH3 and DAPI after treating with all-thiol or disulfide type of HMGB1 confirmed this differential kinetics of two types of HMGB1 (Fig. 3e). These differential potencies were further confirmed in PMNs prepared from bone marrow (Additional file 1: Figure S2). Pretreatments of purified neutrophils with Cl-amidine (a PAD inhibitor, 10 μM) for 30 min clearly suppressed CitH3 inductions by all-thiol and by disulfide HMGB1 (Figs. 3 f and g). Furthermore, pretreatments of purified neutrophils with AMD3100 or TLR4-IN-C34 (antagonists of CXCR4 and TLR4, respectively) suppressed CitH3 induction by all-thiol and by disulfide HMGB1 (Figs. 3f and g), which showed the two HMGB1s induced NET formation via specific receptors.

### Suppressions of MCAO-induced CitH3 induction and NETosis by anti-HMGB1 antibody or a HMGB1 antagonist

**Fig. 4:**
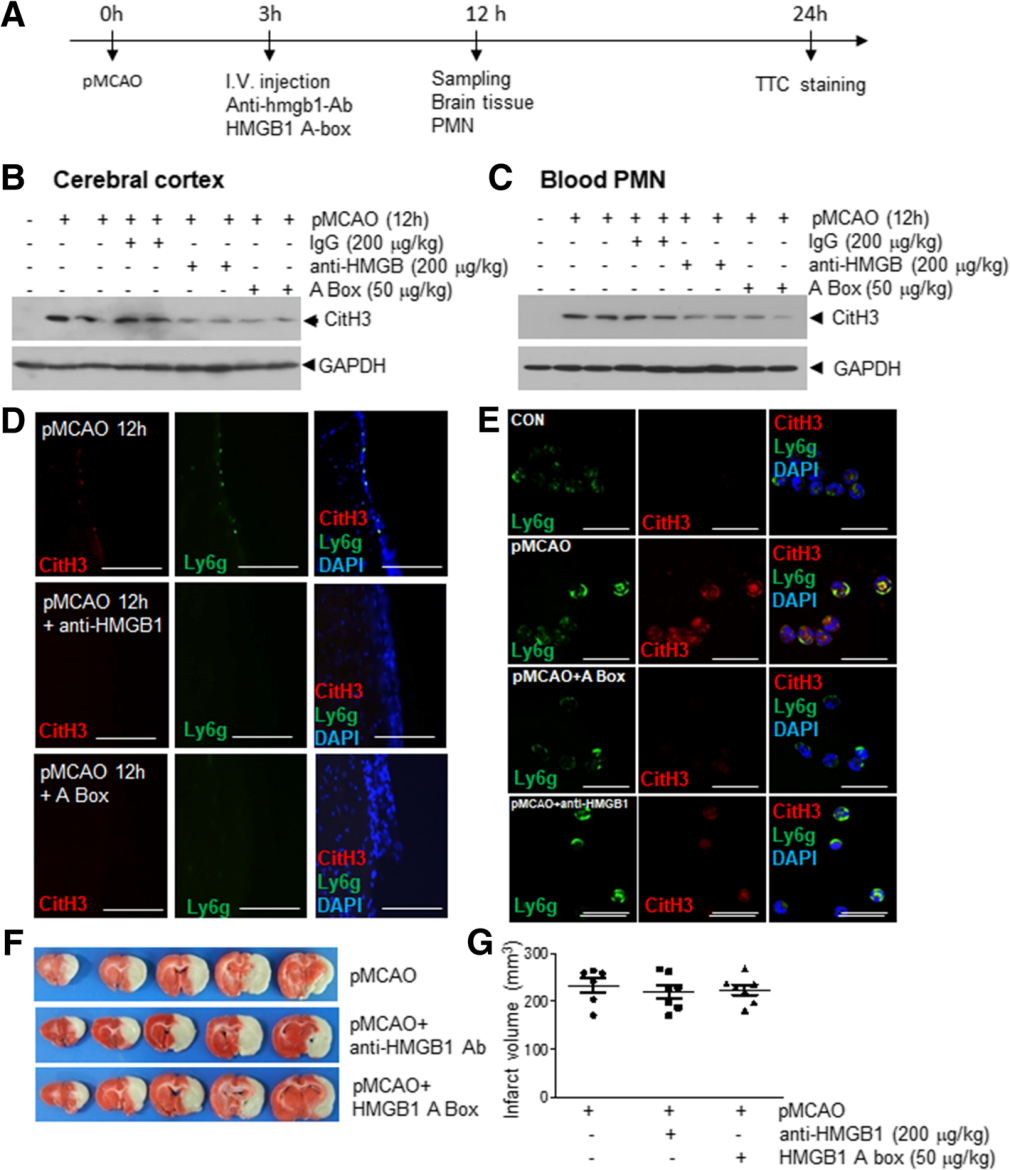
Suppression of CitH3 induction in brain parenchyma and in the peripheral blood after MCAO by anti-HMGB1 antibody or HMGB1 A box. **a** Anti-HMGB1 antibody (200 μg/kg), HMGB1 A box (50 μg/kg), or IgG (200 μg/kg) were administered intravenously after 3 h of MCAO. **b-c** Levels of CitH3 in the cortices of ischemic hemispheres after 12 h of MCAO (**b**) and in neutrophils isolated after 12 h of MCAO (**c**) were examined by immunoblotting. GAPDH was used as a loading control. **d-e** Coronal brain sections (**d**) and blood neutrophils (**e**) were prepared after 12 h of MCAO and triple immunofluorescent staining was conducted using anti-CitH3 antibody, anti-Ly6g antibody, plus DAPI. Scale bars in D represent 100 μm and those in E represent 10 μm. **f-g** Coronal brain sections were prepared after 24 h of MCAO and mean infarct volumes were determined by TTC staining (**f**) and are presented as means±SEMs (**g**). pMCAO, PBS-treated pMCAO control (*n* = 6); anti-HMGB1, anti-HMGB1-treated pMCAO group (*n* = 7); HMGB1 A box, HMGB1 A box-treated pMCAO group (*n* = 7)

To investigate the importance of HMGB1 in NETosis induction in the ischemic brain, we administered anti-HMGB1 antibody or HMGB1 A box (an antagonist of HMGB1) after 3 h of MCAO (Fig. 4a). CitH3 induction observed in cerebral cortices after 12 h were significantly suppressed by anti-HMGB1 antibody and by HMGB1 A box peptide but not by IgG (Fig. 4b). Similarly, CitH3 induction in peripheral blood purified at 12 h was also significantly reduced (Fig. 4c), indicating HMGB1 plays a critical role in inducing NETosis after cerebral ischemia. In addition, triple immunofluorescent staining with anti-CitH3, anti-Ly6g antibodies, plus DAPI revealed significant suppressions of NET formations in cortices of ischemic brains (Fig. 4d) and in PMNs prepared from animals after 12 h of MCAO (Fig. 4e). Since, infarct volumes, measured after 24 h of MCAO, were not significantly reduced in animals administered with HMGB1 antibody or HMGB1 A box (Figs. 4f and g), suppression of NETosis was not an indirect outcome of neuroprotection but rather directly due to blocking HMGB1 function. These results show HMGB1 plays a critical role in the induction of NETosis after MCAO.

### Neuronal cell death induced NETosis and vice versa

**Fig. 5:**
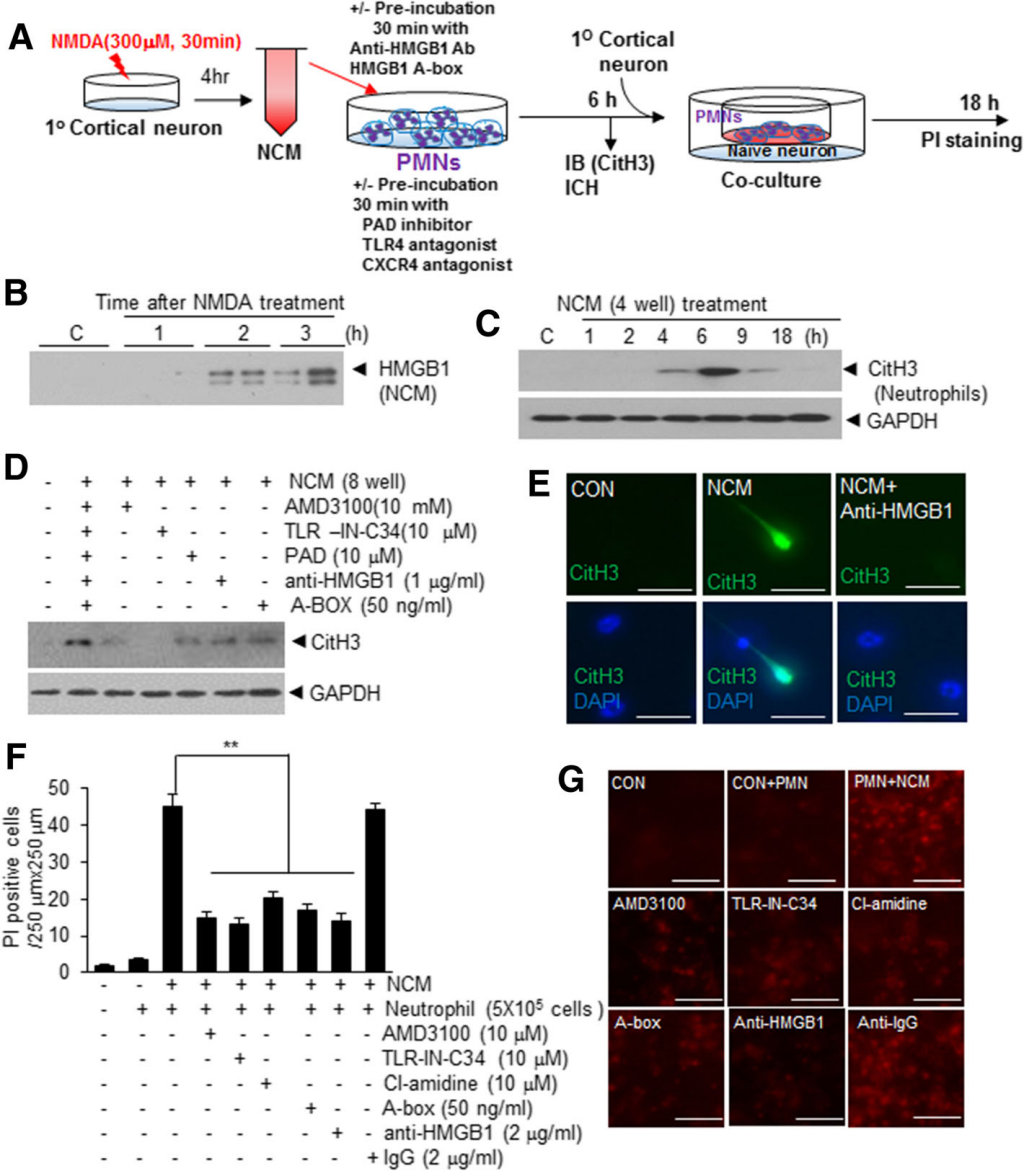
NCM induced NETosis and NETosed neutrophils induced neuronal cell death. **a** Schematic diagram of the procedure used to co-culture NCM-treated neutrophils and primary cortical neurons. **b** HMGB1 levels in NCM of primary cortical neurons (1.6 × 10^6^/4 well) were examined by immunoblotting after 1, 2, or 3 h of NMDA treatment (300 μM, 30 min). **c** Isolated neutrophils (5 × 10^5^/well) were treated with NCM for 1, 2, 4, 6, 9, and 18 h and CitH3 levels were examined by immunoblotting. (D-E) Neutrophils (5 × 10^5^/well) was pre-incubated with AMD3100 (10 μM), TLR4-IN-C34 (10 μM), PAD inhibitor (10 μM) for 30 min and then treated with NCM or was incubated with NCM, which was pre-incubated with anti-HMGB1 antibody (1 μg/ml), HMGB1 A box (50 ng/ml) for 30 min. CitH3 levels in isolated neutrophils (5 × 10^5^/well) (PMNs) were determined by immunoblotting after 6 h (**d**) and NET formation was visualized after 6 h of NCM treatment by double immunofluorescent staining using anti-HMGB1 antibody and DAPI (**e**). **f-g** NCM-treated PMNs (5 × 10^5^/well) were prepared as it was described in D-E and further included IgG (2 mg/ml) control, and then co-cultured with naïve primary cortical culture (4 × 10^5^/well) for 18 h. Numbers of PI-positive cells were counted (250 μm × 250 μm). Scale bars in E represent 20 μm and those G represent 50 μm. Results are presented as means±SEMs. *p < 0.05, **p < 0.0

To further confirm the role played by HMGB1 in NETosis induction in the postischemic brain, we examined the inter-relationship between neuronal cell death and NETosis (Fig. 5a). When primary cortical neuron cultures were treated with NMDA (300 μM, 30 min), HMGB1 was released and accumulated in culture media (Fig. 5b). When these NMDA-conditioned media (NCM) were collected after 4 h of NMDA treatment and treated to neutrophils, marked CitH3 induction was detected after 4 h of treatment and this induction peaked after 6 h (Fig. 5c). However, pre-incubation of NCM with HMGB1 antibody or HMGB1 A box significantly suppressed CitH3 induction, indicating that HMGB1 plays a critical role in NCM-mediated NETosis induction (Fig. 5d). Furthermore, NCM-mediated CitH3 inductions were also inhibited by pre-incubating neutrophils with TLR4-IN-C34 or AMD3100 (Fig. 5d), which indicated TLR4 and CXCR4 were both involved in HMGB1-mediated NETosis induction. NCM-mediated NETosis and the importance of HMGB1 in this process were confirmed by double immunofluorescent staining (Fig. 5e). Furthermore, when these NCM-treated blood neutrophils were co-cultured with naïve primary cortical neurons for 18 h, the number of PI-positive neurons markedly increased, but this increase was significantly reduced when neutrophils were pre-incubated with TLR4-IN-C34, AMD3100, or Cl-amidine before the co-culture or NCM was pre-incubated with anti-HMGB1 antibody or HMGB1 A box and then treated to neutrophils (Fig. 5f and g). However, it was not reduced when neutrophils were pre-incubated with IgG (2 mg/ml) (Fig. 5f and g). These results indicate that HMGB1 play critical roles both in NETosis induction in neutrophils and in NETosed neutrophil-induced neuronal cell death.

### HMGB1 released from neutrophils after NETosis contributed to NETosis-induced neuronal death

**Fig. 6:**
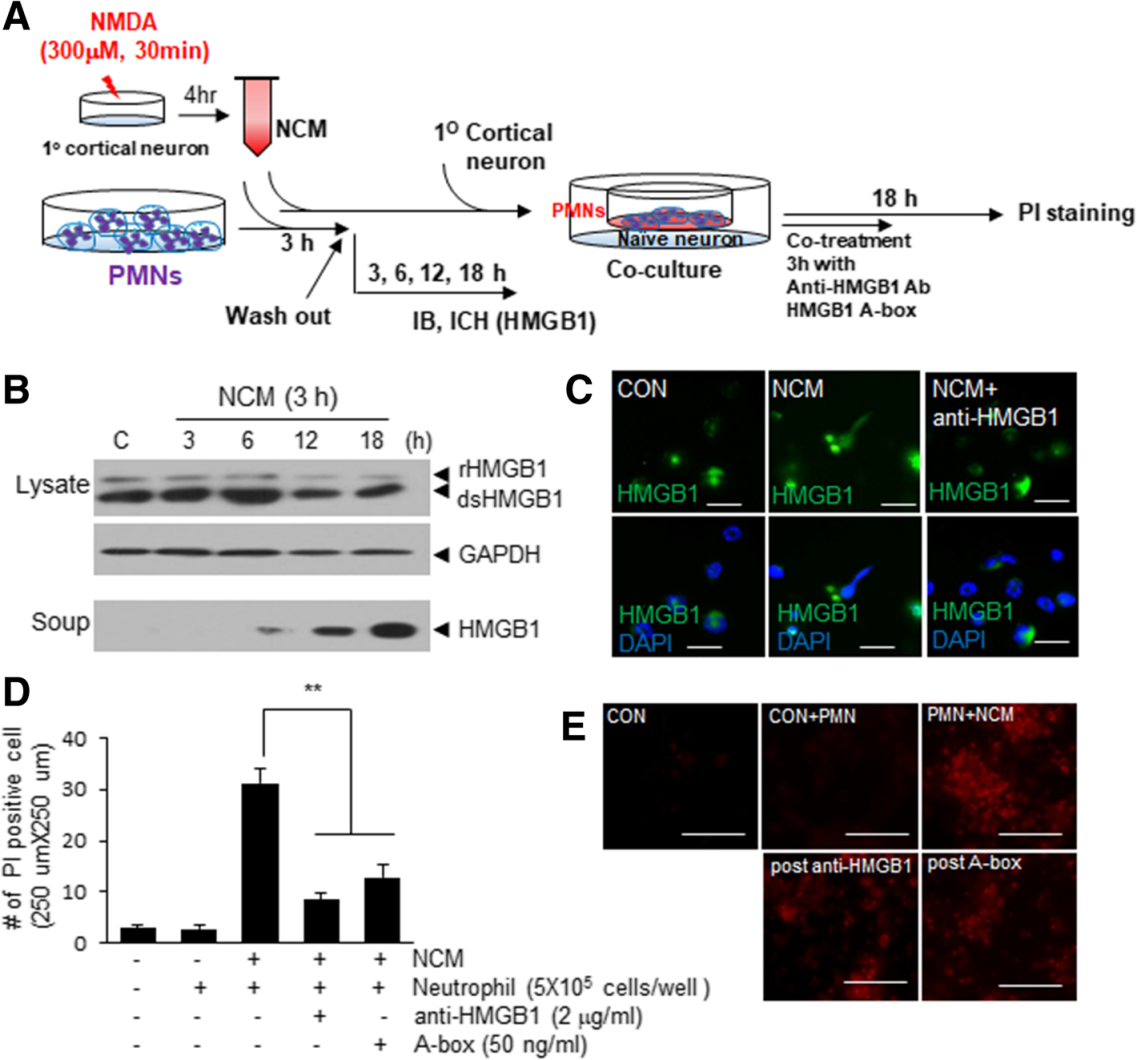
Blocking HMGB1 suppressed NETosed neutrophil-induced neuronal cell death. **a** Schematic diagram of the procedure for blocking HMGB1 in the co-culture of NETosed neutrophils and primary cortical neurons. **b-c** PMNs-BM (5 × 10^5^/well) were treated with NCM (1.6 × 10^6^/4 well) for 3 h and then cultured in fresh media for another 3, 6, 12, or 18 h and HMGB1 levels were examined in cell lysate and culture media by immunoblotting (**b**). NET formation was visualized after 6 h of NCM treatment by double immunofluorescent staining using anti-HMGB1 antibody and DAPI (**c**). **d-e** NCM-treated PMNs were co-cultured with naïve primary cortical culture (4 × 10^5^/well) for 18 h in the presence or absence of anti-HMGB1 antibody (1 μg/ml) or HMGB1 A box (50 ng/ml) and the numbers of PI-positive cells were counted (250 μm × 250 μm). Scale bars in C represent 20 μm and those in E represent 100 μm. Results are presented as means±SEMs. **p < 0.0

To investigate whether HMGB1 is also released from neutrophils after NETosis and contributes to NETosis-induced neuronal death, we examined the levels of neuronal death after blocking HMGB1 during co-culture of NETosed neutrophils and naïve neurons (Fig. 6a). When NETosis was induced by treating circulating neutrophils with NCM, HMGB1 was released from neutrophils and accumulated in cell lysates and culture media (Fig. 6b). In these experiments, to remove the HMGB1 localized in NCM, neutrophil culture media was collected after washing the culture after 3 h of NCM treatment. Localization of HMGB1 immunoreactivity in the extrusions of NETosed neutrophil was confirmed in double immunofluorescent staining (Fig. 6c), which indicated HMGB1 is a component of NETs extruded after NETosis. As it was shown in Fig. 5, when these NCM-treated blood neutrophils were co-cultured with naïve primary cortical neurons, the number of PI-positive neurons increased (Figs. 6d and e). However, when anti-HMGB1 antibody or HMGB1 A box were treated to neutrophil-neuron co-culture for 3 h, the number of PI-positive neurons was also significantly reduced (Figs. 6d and e), indicating HMGB1 released from NETosed neutrophils contribute to neuronal cell death. Taken together, these results reveal a reciprocal aggravating cycle exists between neuronal cell death and NETosis, and that HMGB1 appears to mediate detrimental effects in both directions.

### Inhibition of NET formation mitigated delayed inflammation and vessel damage in the ischemic brain

**Fig. 7:**
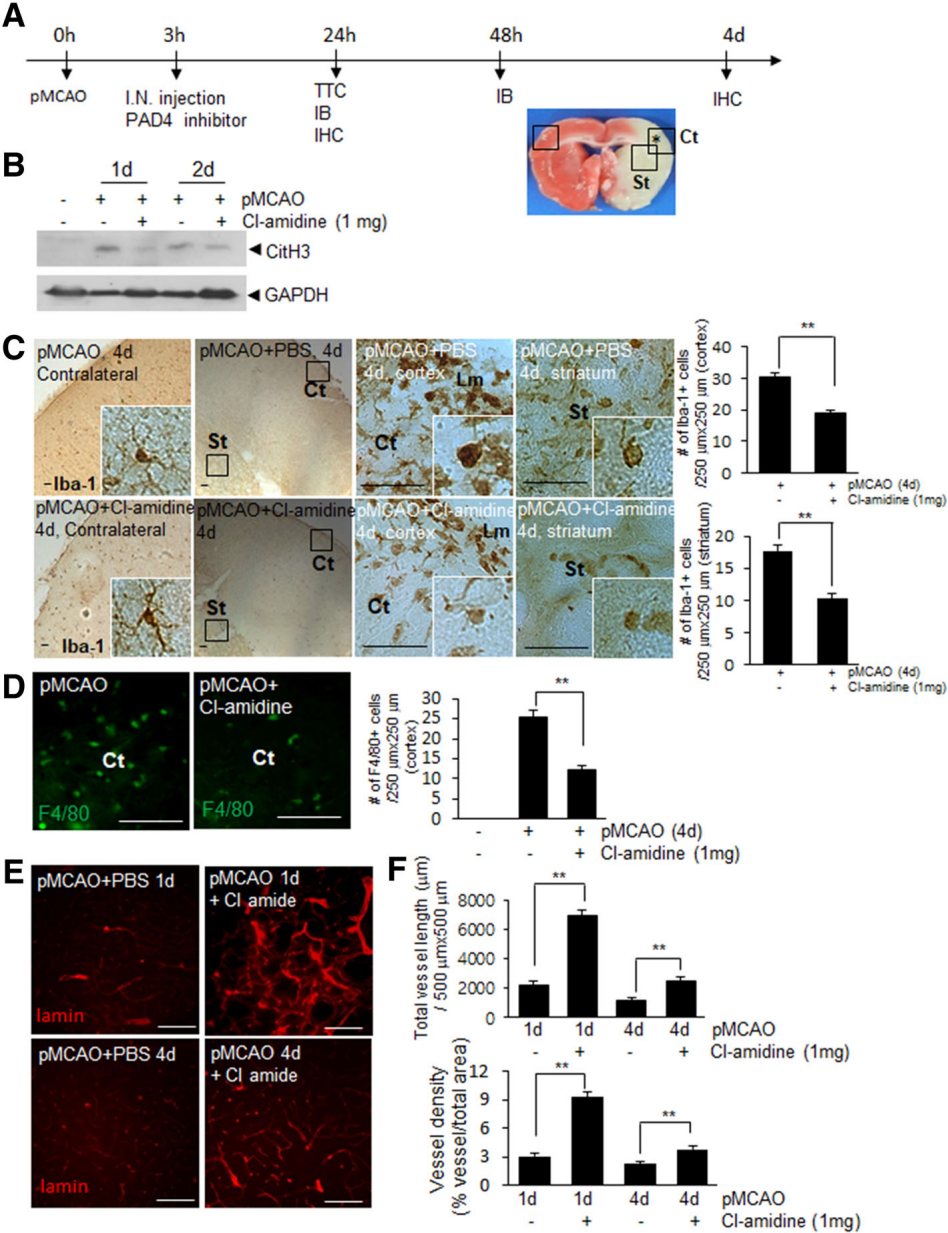
Infiltration of immune cells and vessel damage in the ischemic brain were suppressed by intranasal administration of PAD inhibitor. **a** Cl-amidine (5 mg/kg) was administered intranasally after 3 h of MCAO and levels of CitH3 and numbers and morphology of microglia and blood vessels were examined after 1, 2, or 4 d of MCAO. **b** CitH3 levels in cortices of ischemic hemispheres after 1 or 2 d of MCAO were determined by immunoblotting using GAPDH as a loading control. **c-f** Coronal brain sections were prepared after 4 d of MCAO and stained with anti-Iba-1 (**c**), anti-F4/80 (**d**), or anti-laminin antibody (**e**). Numbers of Iba-1-positive (C, 250 μm × 250 μm) and F4/80-positive cells (D, 250 μm × 250 μm) in cortex (Ct) and striatum (St) were counted and presented as means±SEMs. Total vessel length (E, 500 μm × 500 μm) and vessel density are presented as means±SEMs (F). Scale bars in C represent 200 μm and those in D, E represent 100 μm. Sham, sham-operated animals (*n* = 9 from 3 animals); MCAO+PBS, PBS-treated MCAO control animals (*n* = 12 from 4 animals); MCAO+Cl-amidine, the Cl-amidine-administered MCAO animals (n = 12 from 4 animals). Ct, cortex; St, striatum; Lm, leptomeninges. **p, < 0.01 vs. MCAO+PBS controls

Next, we examined whether suppression of NET formation mitigate the brain damage induced by MCAO (Fig. 7a). When Cl-amidine was administered after 3 h of MCAO, CitH3 induction was significantly suppressed in brain parenchyma examined after 24 or 48 h of MCAO (Fig. 7b). However, reduction of infarct volume was not detected in Cl-amidine-administered MCAO animals (Additional file 1: Figure S3). Importantly, however, immunohistochemical stainings with anti-Iba-1 or anti-F4/80 antibody showed that numbers of microglia and macrophages after 4 d of MCAO were markedly decreased in Cl-amidine-administered MCAO animals both in cortex and striatum and revealed inactivated phenotype of Iba-1^+^ cells (Figs. 7c and d), indicating that inflammation at delayed time points were mitigated. In addition, immunostaining with anti-lamin antibody showed total length and density of blood vessels in cerebral cortex after 1 d of MCAO significantly increased and these protective effects on blood vessel were also observed after 4 d of MCAO (Figs. 7e and f), indicating that inhibition of NETosis suppressed blood vessel damage observed in the ischemic brain. Together these results demonstrate NETosis was related with delayed brain damages, such as, aggravation of inflammation and vessel damage, in our rat model of permanent cerebral ischemia.

## Discussion

The present study shows NETosis was induced in brain parenchyma and peripheral blood in our permanent stroke model and that HMGB1 plays an important role in these processes. It has been reported neutrophil influx is more prominent after permanent than after transient MCAO [3, 27], and in the present study, we also found the frequency and intensity of NETosis were significantly greater after permanent MCAO (data not shown), which suggests a relation exists between NETosis and disease severity. Moreover, in a study of the plasma of acute ischemic stroke patients, it was observed NET marker levels were correlated with stroke severity at onset and discharge from hospital as evaluated using NIHSS and mRs scores, and that elevated levels of CitH3 at onset were associated with all-cause mortality at one-year follow-up visits [36]. Thus, it appears NETosis might be a useful prognostic maker, especially in acute and severe ischemic stroke.

In terms of the spatio-temporal progression of NETosis after permanent MCAO, we first observed neutrophils undergoing NETosis in peripheral blood and leptomeningeal vessels after 12 h of MCAO, and subsequently, their infiltration into brain parenchyma in cortex and striatum after 1 d of pMCAO. Although reports have been issued on the route of neutrophil migration and their activation (NETosis) at 1 d after permanent MCAO in mice [6, 27], this is the first report to describe the temporal and spacial progressions of NETosis after MCAO using an intraluminal model. Furthermore, it should be noted neutrophil recruitment into damaged brain tissue occurred from the stagnated blood in ischemic brain tissues after MCAO and also from the peripheral blood from 12 h after MCAO. Without reperfusion, the main route of neutrophil infiltration from peripheral blood vessels after brain ischemia follows the route; leptomeningeal vessel → Virchow-Robin space → perivascular space → brain parenchyma.

Interestingly, we found that some neutrophils in blood and the CSF of MCAO animals were already CitH3^+^ before they infiltrated brain parenchyma. CitH3^+^ levels were markedly increased in peripheral blood after 12 h of MCAO, when more than 20% of neutrophils expressed CitH3, and then fell abruptly. Based on these observations, we speculate a portion of early infiltrating neutrophils were of the CitH3^+^/non-lytic form and later adopted the lytic form, and that other neutrophils induced CitH3 and NET formation after infiltrating brain parenchyma. We cannot exclude the possibility that some intravascular neutrophils were already lysed (NETosed), and contributed to endothelial cell and BBB damage. The observed accumulation of HMGB1 in serum as early as 6 h after occlusion (Fig. 3) suggests HMGB1 might play a critical role in the early onset of NETosis within blood vessel. Regarding this, it is interesting to note that NETs were reported to form in the sinusoids of ischemic liver lobes [9] and in the blood vessels of a venous thrombosis mouse model [31]. Recently, presence of CitH3-positive neutrophils in the peripheral blood was detected only 4 h after LPS injection in septic liver disease [24] or only 30 min after LPS injection in endotoxemia model [26], indicating that circulating CitH3 might serve as a reliable biomarker for early detection of endotoxic shock. In the present study, we also observed CitH3-positive neutrophils in CSF at 1–4 days after MCAO (Additional file 1: Figure S1), which suggests CSF might facilitate the movement of activated neutrophils and that the choroid plexus might provide a means of neutrophil entry and exit [28]. Therefore, we hypothesized that rapid NETosis of circulating neutrophils within blood vessels might contribute acute inflammation and subsequent brain damage.

In the present study, we found that all-thiol and disulfide HMGB1 are both capable of inducing NETosis but differential kinetics. In view of the observation that mainly all-thiol HMGB1 was present in ischemic brain at 2 h post-MCAO but both HMGB1 types were present at 1 d post-MCAO in serum of stroke patients [19], we speculate all-thiol HMGB1 might be involved in the rapid recruitment of neutrophils to vessel walls and in the robust surge of NETosis in intravascular and leptomeningeal regions soon after occlusion, and then later both disulfide and all thiol HMGB1 play roles. However, it is highly possible disulfide HMGB1 plays more critical roles after the acute phase, because HMGB1 is oxidized quickly in the extracellular space (the half-life of all-thiol HMGB1 is only 17 min in vitro) [43]. Although, HMGB1-TLR4 has been reported to play important roles in NETosis in animal models of inflammatory liver injury or acute lung injury [9, 32], the potencies and differential functions of different redox forms of HMGB1 have not been investigated. Therefore, we suggest further studies to be conducted to determine the importance of different types HMGB1 and their receptors at different times and locations after MCAO in animal models and in stroke patients in the context of NETosis.

In the present study, we showed HMGB1 is a component of the cellular contents extruded during NETosis, thus co-localized with NETs, indicating extracellular HMGB1 was also provided by NETosis. HMGB1 extruded during NETosis may act as a DAMP, exacerbating inflammatory response in the ischemic brain by further recruiting and activating neutrophils and other immune cells. Regarding this, we confirmed blockage of HMGB1 by HMGB1 A box not only suppressed NMDA-conditioned media-induced NETosis induction but reduced NETosis-induced neuronal death if HMGB1 A box was treated to co-culture of neutrophils undergoing NETosis and neurons. These observations indicate the detrimental effect of HMGB1 released by NETting neutrophils and the presence of vicious cycles involving HMGB1 as a main mediator that aggravate inflammatory response during permanent MCAO. Additional studies are also needed to determine whether and how different HMGB1 redox forms contribute to this cycle and to identify the receptors involved.

## Conclusion

The present study showed NETosis aggravated inflammation and subsequent brain damage. Although inhibiting NETosis failed to reduce infarct volume in the animal model used in this study due to severe and rapid progress of the brain damage, mitigations of delayed inflammation and vessel damage were clearly detected. Therefore, NETosis can be a useful prognostic maker in acute ischemic stroke and targeting NETosis by modulating HMGB1 might provide a multipotent therapeutic strategy to mitigate ischemic brain damage.

## Additional file

Additional file 1: **Figure S1.** CitH3 induction in neutrophils localized in CSF prepared after MCAO. **Figure S2.** CitH3 inductions in bone marrow PMNs by HMGB1 treatment. **Figure S3.** Infarct formation in the ischemic brain was not suppressed by intranasal administration of PAD4 inhibitor. (DOCX 1166 kb)

## Data Availability

The data sets generated during the current study are available from the corresponding author on reasonable request.
