# Supplementary material for: Neutrophil extracellular trap induced by HMGB1 exacerbates damages in the ischemic brain
Source: Acta Neuropathol Commun. 2019 Jun 10;7:11. doi: 10.1186/s40478-019-0747-x (PMC6556959; doi:10.1186/s40478-019-0747-x)
Supplement: Supplementary file 1 — Figure S1. CitH3 induction in neutrophils localized in CSF prepared after MCAO. Figure S2. CitH3 inductions in bone marrow PMNs by HMGB1 treatment. Figure S3. Infarct formation in the ischemic brain was not suppressed by intranasal administration of PAD4 inhibitor. (DOCX 1166 kb) [file 40478_2019_747_MOESM1_ESM.docx]

**SUPPLEMENTARY INFORMATION**

**Neutrophil Extracellular Trap Induced by HMGB1 Exacerbates Damages in the Ischemic Brain**

Seung-Woo Kim^1,2^, Hahnbie Lee^2,3^, Hye-Kyung Lee^2,3^, Il-Doo Kim^2,3^, Ja-Kyeong Lee^2,3*^

^1^Department of Biomedical Sciences, Inha University School of Medicine, ^2^Medical Research Center, ^3^Department of Anatomy, Inha University School of Medicine, Inchon, Republic of Korea

Key words: HMGB1, NETosis, inflammation, MCAO, permanent ischemia

^*^ Corresponding author:

Ja-Kyeong Lee, Ph.D.

Department of Anatomy, ^2^Medical Research Center, Inha University School of Medicine. iinha 100, Nam-Gu, Inchon, 22212, Republic of Korea, Tel, +82-32-860-9893; FAX, +82-32-884-2105; [jklee@inha.ac.kr](mailto:jklee@inha.ac.kr)

**Supplementary materials and methods**

1. **Collection of Cerebrospinal Fluid (CSF)**

CSF collection was performed as previously described (Liu et al., 2008; Nirogi et al., 2009). Briefly, a sham or a MCAO-operated rat was anesthetized by administering ketamine (100 mg/kg) and xylazine (10 mg/kg) intramuscularly and placed on the stereotaxic instrument. The surgical site was cleaned with 10% povidone iodine, followed by 70% ethanol, and a sagittal incision of the skin was made inferior to the occiput. A syringe needle (26 1/2 G, 1 ml) was then inserted 5 mm deep to dura at a 45° angle. Reflux of the CSF was observed and approximately 100 μl of CSF was withdrawn. After adding FBS (10 μl) to CSF (90 μl), 100 ul of CSF was attached to slide using cytospin centrifuge.

1. **Isolation of bone marrow neutrophils**

Bone marrow (BM) cells were harvested from hind limb tibiae and flushed with PBS-BG solution using a 22 G needle syringe. BM cells were collected in a 15 ml polypropylene tube (Sarstedt, Nümbrecht, Germany) and pelleted by centrifugation at 600 g for 10 min at 4°C. Cells were suspended in 45% Percoll (GE Healthcare, Danderyd, Sweden) and layered onto a 50%, 55%, 62%, and 81% discontinuous Percoll gradient. The Percoll solution was centrifuged at 1500 g for 30 min at 4°C and neutrophils between the 81% and 62% Percoll layers were harvested into a new 15 ml polypropylene tube. After one wash with 10 ml PBS-BG at 600 g for 10 min at 4°C, remaining red blood cells were eliminated by centrifuging in Histopaque-1119 (Sigma Chemical, St. Louis, MO) at 1500 g for 20 min at 4°C. Approximately 1~3$x$10^7^ cells were obtained per mouse, and of these 80% were neutrophils (identified by staining nuclei with Turks reagent (Merck, Darmstadt, Germany)). These cells were suspended in alpha MEM (Gibco BRL, Gaithersburg, MD) and used within 12 h.

1. **Infarct volume assessment**

Coronally sectioned (2mm) brain slices were immediately stained with 2% 2,3,5-triphenyl tetrazolium chloride (TTC) (37°C for 15min) and fixed in 4% paraformaldehyde. Infarcted tissue areas were measured using the Scion Image program (Scion Corporation, Frederick, MD). To correct for brain edema following ischemia, measured infarct areas were adjusted with respect to areas in contralateral hemispheres. Infarct volumes were calculated (in mm3) by summing infarct sizes on adjacent tissue sections.

**Figure S1**

**
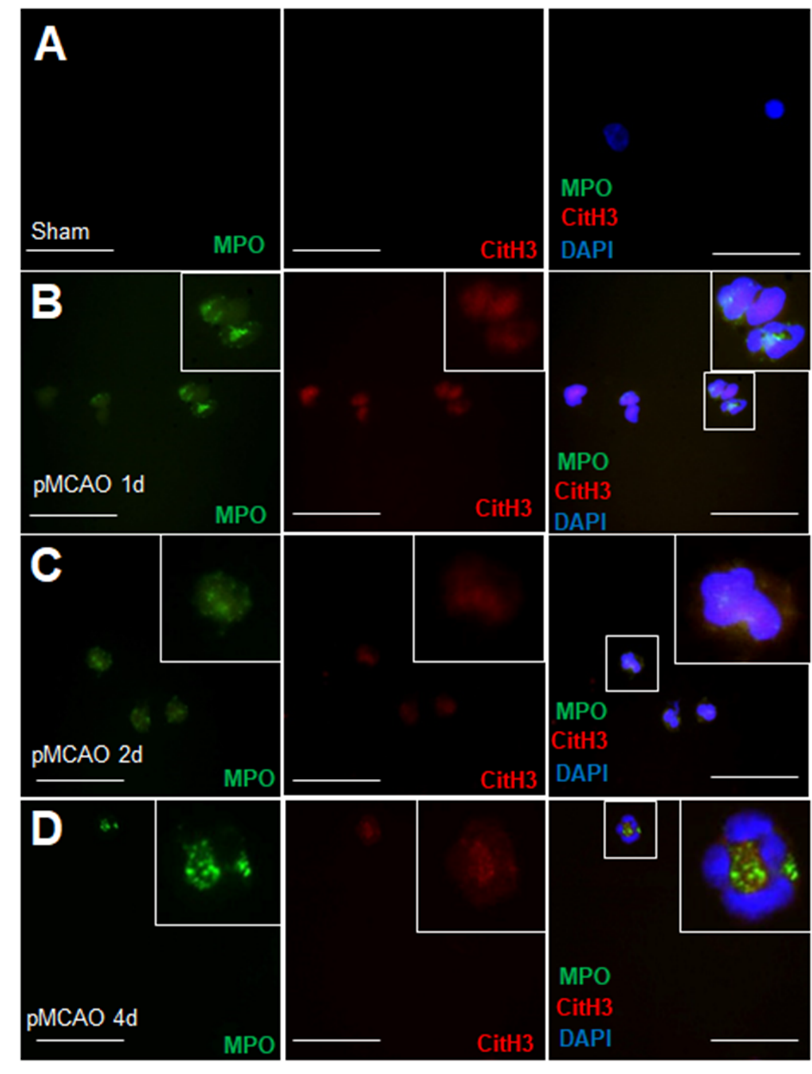
**

**Figure S1. CitH3 induction in neutrophils localized in CSF prepared after MCAO**

CSF was prepared after 1, 2, or 4 d of MCAO and neutrophils were purified. Triple immunofluorescent staining was conducted using anti-CitH3 antibody, anti-MPO antibody, plus DAPI. Scale bars represent 50 μm. The images in the insets are high magnification photographs of each image indicated as white box.

**
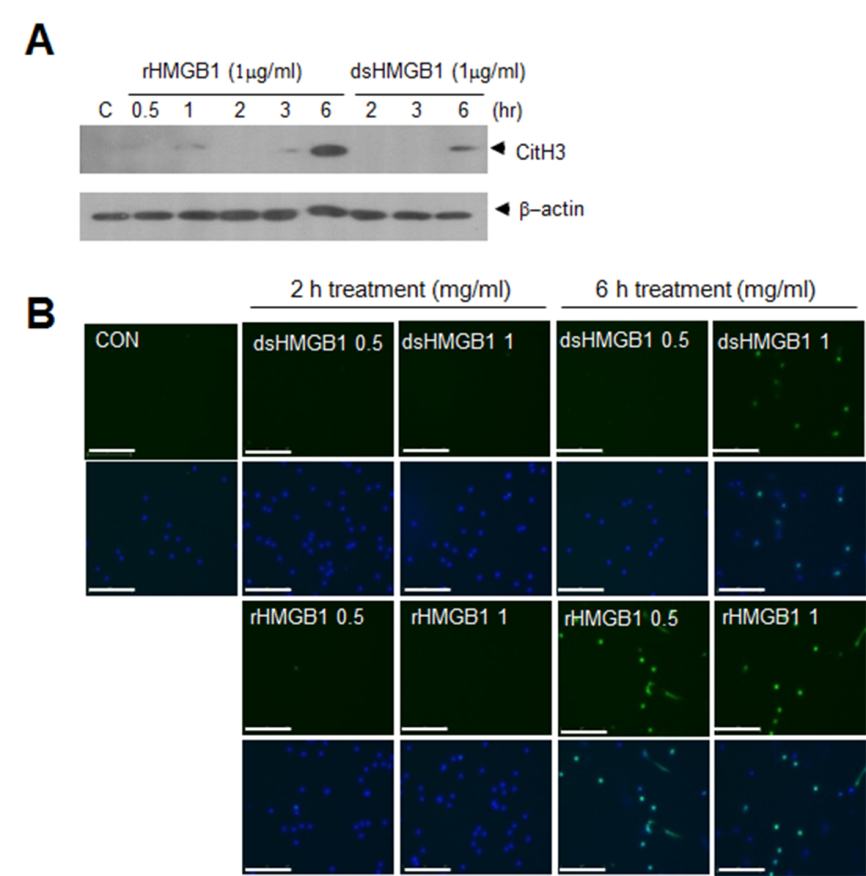
**

**Figure S2.** **CitH3 inductions in bone marrow PMNs by HMGB1 treatment**

(A) PMNs were purified from bone marrow (PMNs- BM) and CitH3 levels were examined after treating them with all-thiol HMGB1 or disulfide HMGB1 (1 μg/ml) for 0.5, 1, 2, 3, and 6 h or for 2, 3, and 6 h, respectively, by immunoblotting. (B) PMNs**-**BM were treated with all-thiol HMGB1 or disulfide HMGB1 (0.5 or 1 μg/ml) for 2 or 6 h and double fluorescent staining was conducted using anti-CitH3 antibody plus DAPI. Scale bars in B represent 125 μm.


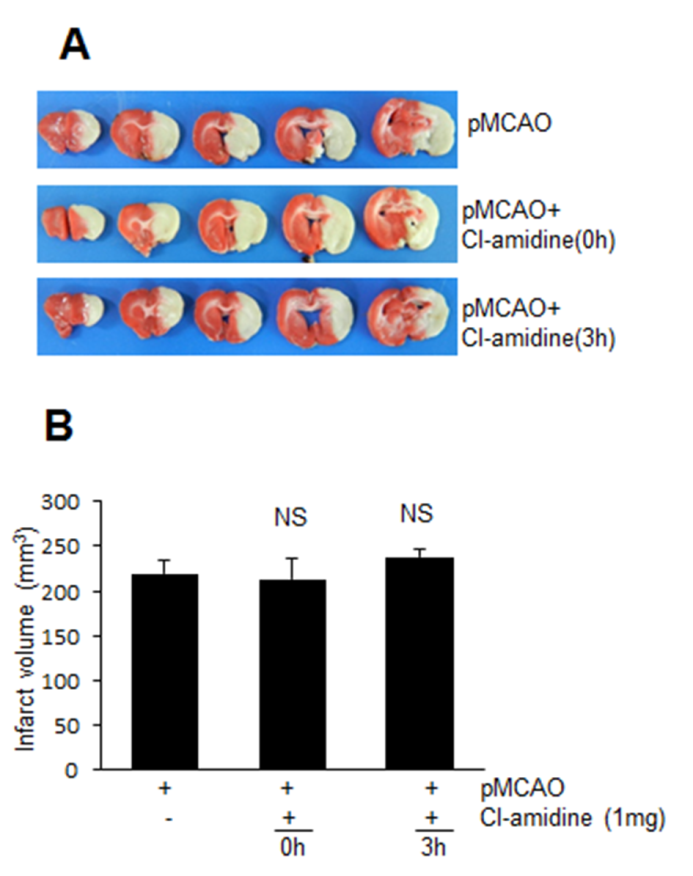


**Figure S3. Infarct formation in the ischemic brain was not suppressed by intranasal administration of PAD4 inhibitor**

Cl-amidine (5 mg/kg) was administered intranasaly right after occulusion or 3 h of MCAO. Coronal brain sections were prepared after 1 d of MCAO and mean infarct volumes were determined by TTC staining (A) and mean infarct volumes are presented as means±SEMs (n=4) (B).
